# Clinical Comparison of the Performance of Two Marketed Ophthalmic Viscoelastic Devices (OVDs): The Bacterially Derived Healon PRO OVD and Animal-Derived Healon OVD

**DOI:** 10.1155/2020/8874850

**Published:** 2020-11-18

**Authors:** Loay Daas, Jose Manuel Larrosa, Alicia Gavin, Carlos Isanta, Achim Langenbucher, Beth E. Jackson, Linda Tsai, Priya Janakiraman, Rafael Guerrero, Berthold Seitz

**Affiliations:** ^1^Department of Ophthalmology, Saarland University Medical Center UKS, Homburg, Saar, Germany; ^2^Hospital Provincial de Nuestra Señora de Garcia, Zaragoza, Spain; ^3^Institute of Experimental Ophthalmology, Saarland University, Homburg, Saar, Germany; ^4^Johnson & Johnson Surgical Vision Inc., Santa Ana, CA, USA

## Abstract

This clinical investigation compared the clinical performance of two marketed ophthalmic viscoelastic devices (OVDs): the bacterially derived Healon PRO OVD (test) and the animal-derived Healon OVD (control) under normal use conditions during cataract removal and lens implantation. This prospective, multicenter, randomized, parallel, participant/evaluator masked, postmarket investigation enrolled 139 subjects (170 eyes), 116 (143 eyes) of which were treated (73 test; 70 control group). Both test and control OVDs were used, at a minimum, to inflate the anterior chamber and protect the endothelium prior to cataract extraction according to the standard procedure. The surgeon completed a postsurgery OVD clinical performance questionnaire, and intraocular pressure (IOP) was measured before surgery and at the 1 day postoperative visit with Goldmann applanation tonometry. Any IOP measurement of 30 mmHg or higher was considered a “spike” and recorded as a study-specific, serious adverse event. The bacterially derived Healon PRO OVD was found to be statistically noninferior to the overall clinical performance of the animal-derived Healon OVD control; thus, the primary hypothesis was satisfied. There were no statistically significant differences between OVD groups for any of the additional endpoints relating to IOP changes or to safety, thus satisfying additional hypotheses. The Healon PRO OVD showed statistically significant improvements in surgeon ratings for ease of injectability, transparency/visibility, and ease of IOL placement. The safety profile was also similar between OVD groups with regards to serious and/or device-related adverse events, as well as medical and lens findings. The results of this clinical investigation support the safety and effectiveness of the bacterially derived, currently marketed Healon PRO OVD and indicate that the intraocular surgical performance was similar between the two OVDs.

## 1. Introduction

Viscoelastics are indicated for use as a surgical aid in anterior segment procedures, including cataract surgery with or without an intraocular lens (IOL), secondary IOL implantation, corneal transplant surgery, and glaucoma filtration surgery [1–4]. Some established benefits of using viscoelastic materials in cataract surgery are endothelial cell protection and maintenance of the intraocular space [1]. The protection provided by viscoelastics results in reduced trauma to the cornea from inadvertent touch due to operative debris from phacoemulsification or surgical instruments during surgery. Minimizing corneal trauma reduces the incidence of endothelial cell loss [5–11] and potential corneal oedema. Viscoelastics also impact the ease of injectability and placement of the IOL as intraocular visibility during surgery is affected by the quantity of bubbles within the OVD. The ease of viscoelastic removal affects both surgical tissue manipulation [12–14] and postoperative IOP [15–21].

Traditional viscoelastics, such as the Healon ophthalmic viscoelastic device (OVD), are developed from sodium hyaluronate derived from rooster combs. Due to concerns of crossover contamination, animal-derived products have been losing favor in many parts of the world. The ophthalmic OVD under investigation (Healon PRO OVD) is a bacterially derived sodium hyaluronate version, formulated to have similar physical properties as the animal-derived version (Healon OVD). This clinical study compared the clinical performance of the two OVDs under normal use conditions during the cataract surgical procedure.

## 2. Materials and Methods

### 2.1. Trial Design

This 2 day, prospective, multicenter, randomized, parallel, double-masked comparative study was an evaluation of user acceptance rates of the bacterially derived Healon PRO OVD (test) versus the animal-derived Healon OVD (control). Both are considered high-molecular-weight cohesive viscoelastics and are manufactured by Johnson & Johnson Vision, Inc.

The study was conducted at two EU sites (Germany and Spain), and 16 surgeons participated in the study and included 170 eyes, each randomized independently to one of the two OVD groups in a 1 : 1 ratio. Participants were implanted with any intraocular lens type and could include one or both eyes in the study.

### 2.2. Subjects

Potential participants had to satisfy the following criteria in, at least, one eye to be enrolled in the study: cataract requiring extraction and posterior IOL implantation and otherwise clear intraocular media. They need to be willing to comply with examination procedures and to sign the IEC-approved informed consent. Exclusion criteria included pupil abnormalities, unresolved ocular surgery, or trauma affecting visual outcomes or increasing ocular risk. Known steroid response, ocular hypertension of ≥20 mmHg, medically controlled ocular hypertension, or glaucomatous changes in the optic nerve were also exclusion criteria, as were pregnancy, lactation, or hormone fluctuation with potential refractive changes. Exclusion criteria also included concurrent participation in any other clinical trial (or within 45 days prior to the preoperative visit).

### 2.3. Procedures

Sequential randomization according to the study randomization log took place after the participant had signed the informed consent document and met all inclusion and exclusion criteria. The investigator documented which eye would be the first to undergo surgery, if applicable, and all study personnel concerned with data collection were masked to the randomization log.

During surgery, masking was maintained by removing the packaging from the randomly assigned OVD out of view of the surgeon prior to use for inflating the anterior chamber and coating the endothelium.

Lens removal was performed using phacoemulsification/aspiration with or without laser fragmentation, and the surgeon's routine choice of commercially available IOL was implanted prior to complete removal of the OVD. Standardization of the surgical technique across study sites was ensured by using routine, small-incision cataract extraction with optic/haptics placed in the capsular bag and sutureless incision closure. Laser-assisted cataract surgery and AK or LRI were allowed bilaterally, any surgical complications were documented, and implantation of an IOL was at the investigator's discretion.

A validated ethics committee-approved questionnaire was completed by the surgeon at the end of each surgical case to collect investigator acceptance for the study OVD used, and the question “Rate the overall performance of the OVD” was used to support the primary objective of the study. Additionally, this questionnaire collected information regarding the ease of injectability, quantity of bubbles, transparency/visibility, tissue manipulation, ease of IOL placement, and ease of removal. Each question was rated on a 5-point scale (1 = unacceptable, 3 = acceptable, and 5 = excellent). Consistency was ensured by using the same study evaluator for all pre- and postoperative study-related subject testing.

Medications were administered as consistent with the investigators routine medication regimen.

The operative visit supplied the primary endpoint: rate of user acceptance as reported via surgeon questionnaire response.

Other endpoints gathered included the following:

1. The rate of IOP spikes, defined as 30 mmHg or greater at 1 day postoperatively as measured by Goldmann applanation tonometry
2. Mean change in IOP at 1-day postoperative visit from baseline
3. Frequency and proportion of each slit-lamp finding
4. All serious or device-related adverse events

### 2.4. Statistical Analysis

The required sample size was calculated based on the percentage of eyes with acceptable overall performance of the OVD (defined as having a score of 3, 4, or 5; 3 = acceptable and 5 = excellent). The acceptable overall performance rate was assumed to be 85% for both test and control OVDs. With 71 eyes per group, the study had 80% power to evaluate the surgeon's acceptance rate of test eyes as being no more than 15% worse than the surgeon acceptance rate of control eyes, using one side of a two-sided 90% confidence interval. Adding 20% for subjects lost to follow-up and screen failure yielded a sample size of 85 eyes per group (170 eyes for the study).

Descriptive statistics included sample size (N), mean, standard deviation (SD), median, minimum (Min.), and maximum (Max.) as appropriate for continuous variables, and frequency and proportion were computed for categorical data.

For continuous variables, statistical tests assuming normality were generally used. Two-sample *T*-tests were used for continuous data, and the Wilcoxon rank sum test was used for ordinal data. The primary endpoint uses a noninferiority method and was evaluated using a one-sided alpha of 0.05 (with a two-sided 90% confidence interval of the difference), and the rest of the endpoints used an alpha of 0.05 for two-sided statistical testing. All available data (Safety Population, SP) were used for the data analysis.

The primary endpoint was the rate of user acceptance defined as having a response of “3, 4, or 5” in overall clinical performance by the surgeon to the study viscoelastic questionnaire. The rate was reported by OVD groups, and the differences (test minus control) were analysed using a noninferiority margin of 15%. The lower confidence interval of the two-sided 90% confidence interval (equivalent to the 1-sided 95% CI) was used for evaluation, and the null hypothesis was that the test OVD was 15% worse than or equal to the control OVD. The alternative hypothesis was that the test OVD was not 15% worse than or equal to the control OVD.

Other endpoints were analysed and reported by the OVD group, including the frequency and proportion of

1. responses to each of the other investigator questionnaire questions
2. eyes having a postoperative IOP spike
3. minimum and maximum change of IOP from baseline (1-day postop IOP minus preop IOP; the preop visit was within 45 days of surgery)
4. Slit-lamp findings

## 3. Results

### 3.1. Participant Demographics

A total of 139 subjects (170 eyes) were enrolled, 116 (143 eyes) of which were treated, 73 eyes received the bacterially derived Healon PRO OVD (test group) and 70 eyes received animal-derived Healon OVD (control group). Of the remaining 27, 15 were screen failures and 12 were discontinued. The 12 discontinued subjects were discontinued for the following reasons: the subject was uncooperative/refused further participation (*N* = 7), the surgeon did not use the study OVD (*N* = 4), and another surgeon not in the study performed the surgery (*N* = 1). The mean patient age was 72.1 years (range: 54–94 years), 56% were female, and all participants were Caucasian. There were no statistically significant differences in the demographics between the Healon PRO and Healon OVD groups.

Participant baseline ocular clinical characteristics/medical findings were evaluated by slit-lamp biomicroscopy; the findings and percentages in the two groups were similar. Findings included blepharitis/meibomianitis, controlled diabetes without retinopathy, dry eye, lids/adnexa (dermatochalasis, trichiasis, and ptosis), vitreous detachment and/or floaters, corneal findings, and retina findings.

### 3.2. Questionnaire Results

**Figure 1:**
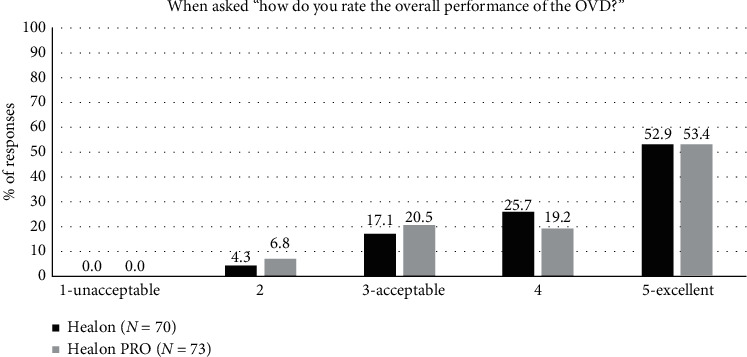
Rate of the overall clinical performance of the Healon PRO OVD (test) and Healon OVD (control), “acceptable” having a response of “3, 4, or 5” in the overall clinical performance of the study viscoelastic.

**Figure 2:**
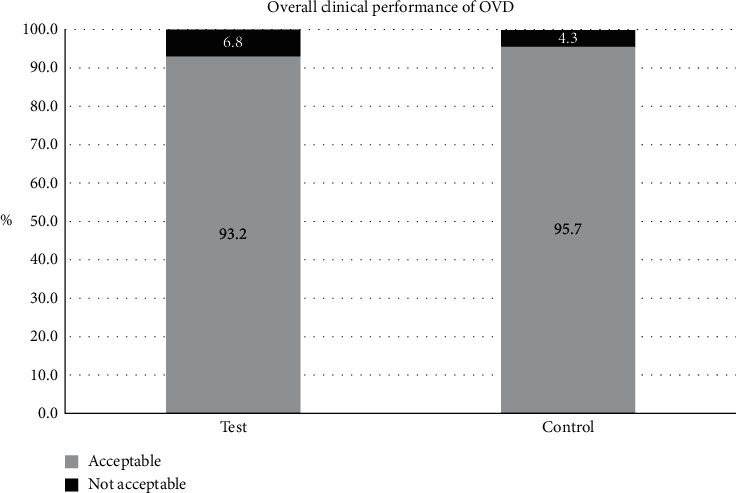
Overall clinical performance of the Healon PRO OVD (test) and Healon OVD (control), “acceptable” having a response of “3, 4, or 5” in the overall clinical performance of the study viscoelastic.

The primary endpoint related to “the overall performance of the OVD” was obtained from the investigator questionnaire using a 5-point scale (1 = unacceptable, 3 = acceptable, and 5 = excellent). The rates of user acceptance (“acceptable-excellent”) were 93.2% for the Healon PRO OVD and 95.7% for the Healon OVD (Figures 1 and 2).

The null hypothesis was that the user acceptance rate of the Healon PRO OVD would be 15% worse than or equal to that of the Healon OVD. The success criterion is defined as having the lower 2-sided 90% confidence interval of the difference in ratings between OVDs be greater than −15%. The difference in user acceptance rates between the two groups was −2.59% (90% CI, −8.85% to 3.72%). The lower CI of the difference was −8.85%, which was higher than the noninferiority margin of −15%; thus, the null hypothesis was rejected, and it was concluded that the Healon PROV OVD was not statistically noninferior in the overall clinical performance compared to the Healon OVD.

**Figure 3:**
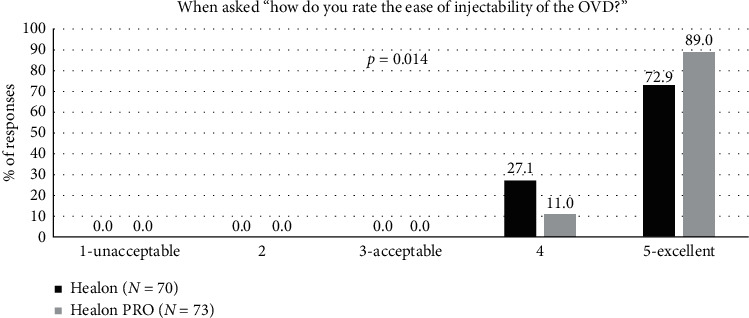
Ease of OVD injectability during surgery for the Healon control and Healon PRO test groups, where “user acceptable” indicated a response of “3,” “4,” or “5.”

The investigator questionnaire also provided data on the following variables: Ease of injectability was given high ratings by the surgeons for both OVDs, with a majority of the responses rated as “excellent”: 89.0% for the Healon PRO OVD and 72.9% for the Healon OVD (Figure 3). There was a statistically significant difference between OVD groups, favouring the Healon PRO OVD (*p*=0.0134) The *p* values for the questionnaire endpoints are from the two-sided Wilcoxon rank sum test.

**Figure 4:**
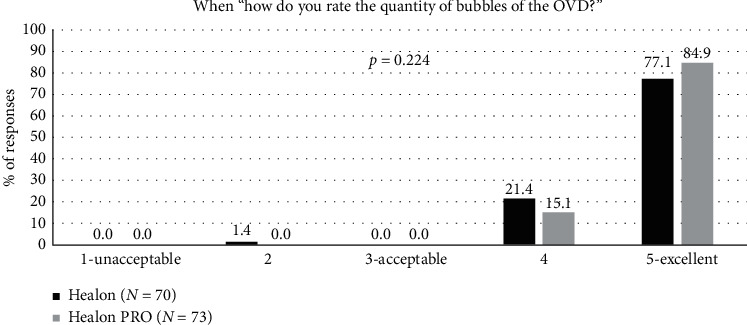
Quantity of bubbles during surgery for the Healon control and Healon PRO test groups, where “user acceptable” indicated a response of “3,” “4,” or “5.”

Quantity of bubbles was rated as “excellent” in most instances for both OVDs: 84.9% for the Healon PRO OVD and 77.1% for the Healon OVD control (Figure 4). Because bubbles obscure the surgeon's view, having a smaller quantity of bubbles was preferred. There was no statistically significant difference in the quantity of bubbles (*p*=0.224) between the two OVDs.

**Figure 5:**
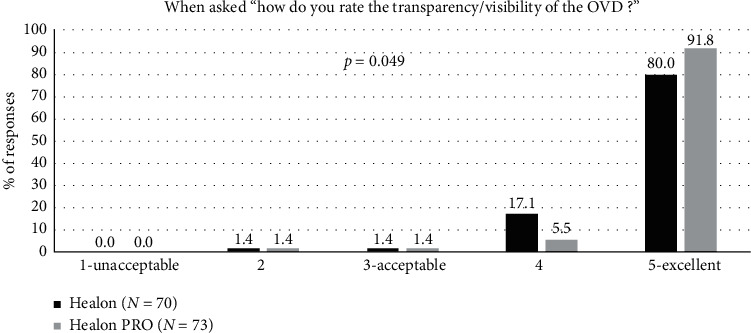
Transparency/visibility during surgery for the Healon control and Healon PRO test groups, where “user acceptable” indicated a response of “3,” “4,” or “5.”

Transparency/visibility was rated as “excellent” in a majority of cases for both OVDs: 91.8% for the Healon PRO OVD and 80.0% for the control (Figure 5). There was a statistically significant preference in transparency/visibility for the Healon PRO OVD when compared to the Healon OVD (*p*=0.049).

**Figure 6:**
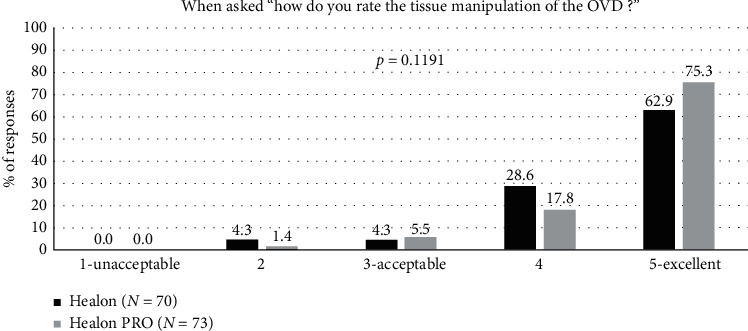
Tissue manipulation during surgery for the Healon control and Healon PRO test groups, where “user acceptable” indicated a response of “3,” “4,” or “5.”

Tissue manipulation ratings were high for both OVDs, with over 95% of ratings being acceptable (Figure 6). There was no statistically significant difference in the scores between the two groups for tissue manipulation (*p*=0.119).

**Figure 7:**
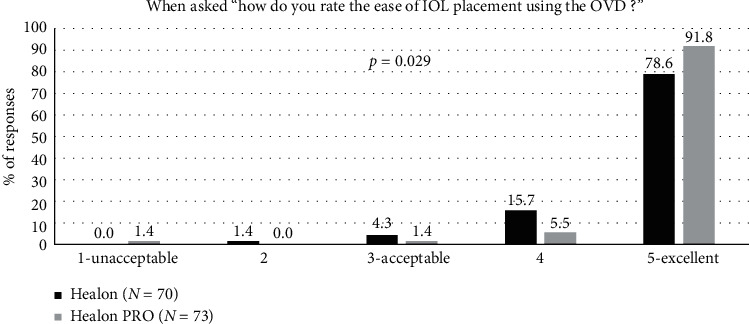
Ease of IOL placement during surgery for the Healon control and Healon PRO test groups, where “user acceptable” indicated a response of “3,” “4,” or “5.”

Ease of IOL placement was rated “excellent” in 90% of the ratings for the Healon PRO OVD, with a statistically significant improvement in ease of IOL placement between the OVD groups favouring the Healon PRO OVD (*p*=0.029) (Figure 7).

**Figure 8:**
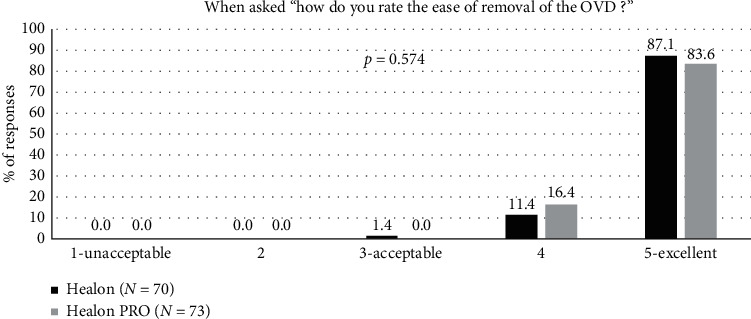
Ease of removal of the OVD during surgery for the Healon control and Healon PRO test groups, where “user acceptable” indicated a response of “3,” “4,” or “5.”

Ease of removal was rated acceptable in all cases for both OVDs, with a majority being rated “excellent” (Figure 8). There was no statistically significant difference in the ease of removal (*p*=0.574).

### 3.3. Other Endpoints

The rate of IOP spike, defined as 30 mmHg or greater at 1 day postoperatively, revealed no statistically significant difference (*p*=0.999) (from two-sided Fisher's exact test). The IOP spike rates for the Healon PRO OVD and Healon OVD were 5.5% and 4.3%, respectively.

**Figure 9:**
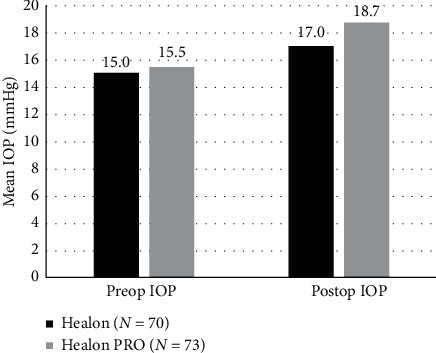
Mean change in IOP measured at baseline and at 1 day postoperatively.

The mean differences in IOP from baseline to 1 day postoperatively were 3.2 mmHg (95% CI, 1.8 to 4.6) for the Healon PRO OVD and 2.0 mmHg (95% CI, 0.9 to 3.2) for the Healon OVD (Figure 9). The mean difference between OVDs was −1.1 mmHg (95% CI, −3.0 to 0.7), with no statistically significant difference in the change of IOP between the Healon PRO OVD and Healon OVD (*p*=0.219 from two-sided two-sample *t*-test).

### 3.4. Ancillary Analyses

Surgical complications were rare, with only one complication (1.4%, 1/73) of zonular damage/rupture occurring in the Healon OVD group and one complication (1.4%, 1/70) of vitreous loss occurring in the Healon PRO OVD group. No surgical procedures outside of the protocol were performed.

The most common adverse event reported was elevated IOP, and this was similar between groups: 6.8% (5/73) in the Healon PRO OVD group and 4.3% (3/70) for the Healon OVD control group.

## 4. Discussion

Viscoelastics are indicated for use as a surgical aid in anterior segment procedures [1–3, 4] and have an important protective role in minimising corneal trauma [5–11]. This clinical study compared the clinical performance of the two OVDs under normal use conditions during the cataract surgical procedure and addresses the trend of moving from animal- to bacterially derived sodium hyaluronate for use in OVDs.

This 2 day, prospective, multicenter, randomized, participant/evaluator-masked clinical investigation evaluated the acceptability of the bacterially derived Healon PRO OVD when compared to the animal-derived Healon OVD control under normal use conditions during the cataract surgical procedure. The primary hypothesis was that the overall clinical performance of the test product would be evaluated by the surgeon to be statistically noninferior to the control group. The results show that the Healon PRO OVD was statistically noninferior to the Healon OVD in the ratings for the overall clinical performance, and thus, the primary hypothesis was achieved and the null hypothesis rejected. Whilst the rates of user acceptance (“acceptable”–“excellent”) were high for both the Healon PRO OVD and for Healon OVD, with a majority of the ratings being “excellent,” these results indicate that the Healon PRO OVD is an appropriate alternative to the animal-derived Healon OVD.

The use of viscoelastics aids the manipulation of surgical tissue [12–14] and contributes to the stability of postoperative IOP [15–21]. The Healon PRO OVD showed statistically significant superiority when compared to the Healon OVD in surgeon ratings for ease of injectability, transparency/visibility, and ease of IOL placement. These factors may prove particularly valuable in the implantation of complex IOLs, in nonstandard eyes, and in securing the reliability and predictability of all anterior eye surgeries. During surgery, bubbles obscure the surgeon's view; hence, a smaller quantity of bubbles is preferred. The study revealed no statistically significant difference in the quantity of bubbles between OVD groups or when surgeons rated the OVDs for ease of tissue manipulation or ease of removal. All ratings were graded high, with either statistical superiority or no statistical difference between the two OVDs, again supporting Healon PRO OVD as an appropriate alternative to the Healon OVD.

Intraocular pressure spikes at the 1 day postoperative visit showed no statistically significant difference between Healon PRO and Healon OVDs. The rate of IOP spikes were comparable with those previously reported for other OVDs. In addition, there was no statistically significant difference in the mean change of IOP from baseline to 1 day between the Healon PRO OVD and Healon OVD groups. The safety profile was similar between OVD groups with regards to serious and/or device-related adverse events, medical findings, and lens findings. There were no statistically significant differences between OVD groups for the occurrence of surgical complications or additional surgical procedures.

Potential limitations of this clinical study should be taken into consideration when interpreting these findings. First, the focus of the study was on qualitative not quantitative data; the key findings from this study were obtained from surgeon-rated responses to questionnaires. Additional data could, perhaps, have been gathered by calculating the time taken on the different procedures or by including refractive outcomes as a measure of success. Second, results may have varied between surgeons/surgical technicians, but restricting the site number to two and ensuring that only fully-protocol-trained staff were included have helped to minimize this random error. Finally, this trial was conducted in the EU, and all of the participants were Caucasian, thus limiting intersubject variety.

In conclusion, this study has demonstrated that the overall clinical performance of the Healon PRO OVD was noninferior to that of the Healon OVD. All study endpoints were achieved, and results were comparable between OVD groups. Hence, the results of this clinical investigation support the safety and effectiveness of the bacterially derived, marketed Healon PRO OVD.

## Data Availability

The data used to support the findings of this study are available from the corresponding author upon request.
